# School mental health promotion in Indonesia: a quantitative survey from Surabaya

**DOI:** 10.3389/fpsyg.2025.1680302

**Published:** 2026-02-16

**Authors:** Margaretha Margaretha, Peter S. Azzopardi, Jane Fisher, Susan M. Sawyer

**Affiliations:** 1 Department of Paediatrics, Melbourne’s Children, Faculty of Medicine, Dentistry and Health Sciences, University of Melbourne, Melbourne, VIC, Australia; 2 Centre for Adolescent Health, Royal Children’s Hospital, Melbourne, VIC, Australia; 3 Murdoch Children’s Research Institute, Melbourne, VIC, Australia; 4 Faculty of Psychology, Universitas Airlangga, Surabaya, East Java, Indonesia; 5 Adolescent Health and Wellbeing, Telethon Kids Institute, Adelaide, SA, Australia; 6 School of Public Health and Preventive Medicine, Faculty of Medicine, Nursing and Health Sciences, Monash University, Melbourne, VIC, Australia

**Keywords:** mental health promotion, implementation, barriers, enablers, schools, Indonesia

## Abstract

**Introduction:**

Most young people live in low- and middle-income countries (LMICs), yet little is known about the implementation of school mental health promotion strategies in resource-poor settings. This study describes the extent of school mental health promotion and its drivers in junior high schools in Surabaya, Indonesia.

**Methods:**

Data were obtained from a sample of 161 schools (63 public schools (100% participation), 74 private schools (28.79% participation), and 24 madrasas (42.85% participation)) using an online survey supported by the Department of Education. Descriptive statistics (ANOVA and Chi-square tests) were used to analyse data.

**Results:**

Most programs addressed mental health as part of comprehensive school health strategies derived from national and local policies. Implementation was reported through five key programs: 1) health-promoting school strategies through the school health unit, 2) safe and healthy school environments through child-friendly schools, 3) support through peer counsellors, 4) mental health as an extra-curricular health education module, and 5) mental health risk assessments to inform school interventions. Public schools reported the highest success in implementing these programs. Madrasas scored lower across all programs.

**Discussion:**

While school mental health promotion is acknowledged through national policies, its implementation in Indonesia appears to be challenging. The development of national standards, investment in school leadership, and support for broader collaboration, especially among ministries of education, religion, and health, could enhance the implementation of school mental health promotion in Indonesia.

## Introduction

Mental health problems account for 14% of the Global Burden of Disease (GBD) in 10-19-year-old adolescents, of whom around 90% live in low- and middle-income countries (LMICs) (Kieling et al., 2011; World Health Organization, 2021, 2023; United Nations, 2022). Despite this, adolescent mental health and its determinants remain largely neglected in LMICs (Patel et al., 2008; Fazel et al., 2014). In part, this reflects sparse data as lack of national prevalence estimates in many countries reduces the visibility of mental disorders compared with other disorders (Erskine et al., 2017, 2023). There are also significant knowledge gaps about what strategies, policies and programmes can promote wellbeing and prevent mental disorders in young people (Dick and Ferguson, 2015). Even less is known about the potential role of schools concerning mental health, especially in LMICs, as most school-based studies have been conducted in high-income countries (Murphy et al., 2017; Nagata et al., 2018; Aston et al., 2023).

Indonesia is a highly populous country with around 70 million school-age children aged 5 to 19 years old (Badan Pusat Statistik, 2023). This makes schools an important setting for considering mental health promotion, prevention and access to health services (Sawyer et al., 2025). A recent study by the National Adolescent Mental Health Surveys (NAMHS) found that the prevalence of mental disorders among 10–17-year-old Indonesians in the past 12 months was 5.5% (95%CI 4.3–6.9) (Erskine et al., 2024). Yet like most LMICs, Indonesia is also dealing with a substantial gap between the burden of mental health needs and the capacity of health services to respond (World Health Organization, 2020), as only 2.6% of young people with mental health problems in a national sample had accessed health services for support or counselling (Center for Reproductive Health Universitas Gajah Mada, University of Queensland, and Johns Hopkins Bloomberg School of Public Health, 2022). Similarly, poor mental health literacy is compounded by stigmatisation of mental health issues, limited skilled mental health professionals, and fragmented service delivery models, intensified by low prioritisation and limited resource allocation from governments (Patel et al., 2008; Kieling et al., 2011; Wainberg et al., 2017; Desi et al., 2018; Brooks et al., 2022a). Similar observations have been made in Southeast Asian countries, where the rise in adolescent mental health issues since the COVID-19 pandemic has not been comprehensively addressed (Menon et al., 2025; Szücs et al., 2025). Most mental health interventions in the region focus on individually-based and clinically oriented management responses, and mental health promotion through universal interventions remains underrepresented in Southeast Asia and the Pacific (Chatwiriyaphong et al., 2025; Menon et al., 2025). Beyond health services, these same issues are a challenge for LMICs in prioritising and implementing mental health promotion (Margaretha et al., 2023).

Currently, little is known about the implementation of school-based mental health promotion and its impacts on students in Indonesia. Even though the school mental health curriculum is undergoing progressive improvements, the Indonesian government is still searching for the best framework to address mental health in schools (Tambun, 2020; Willenberg et al., 2020; Pangiras et al., 2021; Subu et al., 2021; Brooks et al., 2022b). The school health system in Indonesia is further complicated by multiple authorities responsible for managing education. Building on an exploratory qualitative study in Surabaya, Indonesia (Margaretha et al., under review^1^), this study aimed to gather empirical evidence on the current implementation of school-based mental health promotion in junior high schools in Indonesia.

## Methods

### Study context

The city of Surabaya, the capital of East Java Province, spans inner urban and suburban areas with families of varying socio-economic backgrounds. Surabaya has 378 Junior high schools (Sekolah Menengah Pertama, SMP) that provide education to 118,849 students aged 11–16 years old (Badan Pusat Statistik Provinsi Jawa Timur, 2018). Junior high schools can be categorised into public and private, as well as Islamic religious schools, known as Madrasah Tsanawiyah (MTs). In total, 107,346 students are listed as studying in junior high schools under the national authority of the Ministry of Education (MoE) and managed through the Department of Education (DoE) at the Municipality of Surabaya. At the same time, 11,503 students are reported to be studying in MTs under the national-level administration of the Ministry of Religion (MoR) and through municipality-level management by the Department of Religion (DoR) of Surabaya Municipality.

### Instrument

An online school survey was used to assess the approach to mental health promotion in schools. The survey items were developed following a qualitative study that explored barriers and enablers to school mental health promotion implementation (Margaretha et al., under review, see footnote 1). That study identified that the Indonesian government was supporting five school health programs deemed relevant to mental health (see Panel 1 for description). The survey consisted of 29 items which included a set of brief questions about the school’s demographic context (e.g., type of school, student numbers, the number of school counsellors) and then explored: (1) frameworks for school mental health promotion (e.g., focus, scope and approach); and (2) implementation strategies (e.g., school roles, enablers, barriers, and support needed to leverage program) (for details, see Supplement A). A 90-min pilot online session was conducted with six teachers and mental health professionals that also obtained face validity.

**PANEL 1:** Key school-based mental health-related programs in Surabaya.

**School Health Unit (Usaha Kesehatan Sekolah; UKS)** Definition: a school health policy that uses a comprehensive approach to school health to improve students’ capacity to live healthy lives in healthy environments. UKS is well-regulated and nationally applied in all schools (primary and secondary). A school health team forms the basis of overseeing UKS, consisting of school staff, including teachers, and government health officials. This team guides the implementation of UKS, which is designed to engage the whole school community and its related stakeholders. UKS strategies to promote school health are mandated through three components: health education, health services, and creating a healthy school environment. Authority: This national mandate for schools is provided through collaboration between the Ministry of Education and Culture, the Ministry of Health, the Ministry of Religion, and the Ministry of Internal Affairs.
**Child Friendly School (Sekolah Ramah Anak; SRA)** Definition: a child protection policy that aims to guarantee children’s right to learn in a safe and secure school, to be assisted by skilled teachers and educational staff, and well-supported with learning resources and a healthy environment. The implementation of SRA requires a designated task force, which consists of representatives from students, parents, teachers, school counsellors, alumni, and the community, appointed by the school. The SRA team is expected to collaborate with its related stakeholders to support implementation. There is national endorsement that SRA must be implemented in all schools and other child-related settings in Indonesia (e.g., Pesantren or Islamic boarding schools, orphanages). Authority: This national policy is driven by the Ministry of Women’s Empowerment and Child Protection (MoWECP).
**Health Education Modules (Rapor Kesehatanku; RK)** Definition: Health education modules that can be used as extra-curricular strategies by teachers, the school health team, and Puskesmas (community health centres). The students also receive a personal health record book. The modules are designed to equip primary and secondary school students with health knowledge, enhancing their capacity to lead a healthy life and minimise risky behaviours. Mental health is one of the seven health topics in the modules, which is delivered as an extra-curricular activity. Authority: This national policy is drive by the Ministry of Health (MoH).
**Peer Counsellors (Konselor Sebaya: KS)** Definition: a group or cadre of youth health leaders that can be involved in many school health programs. Selected student health leaders are chosen to receive training to improve their knowledge about mental health problems, including how to perform basic peer counselling. School counsellors are also trained to mentor peer counsellors. Authority: This local policy is driven by the Department of Education, the Municipality of Surabaya.
**Mental Health Assessment (Jiwa Rokok Narkoba: Jirona)** Definition: a self-report survey completed by students to assess mental health, smoking and substance abuse. This program is recognised as best practice by the Department of Health of the Municipality of Surabaya. In 2011, the Health Department initiated screening for mental health problems in school-age children and adolescents, and developed assessment tools for Kindergarten, Primary schools, Junior high schools, and Senior high schools. It was planned that this screening would be conducted annually through Puskesmas (community health centres). Authority: This local policy is driven by the Department of Health of the Municipality of Surabaya.

### Data collection procedure

Data were collected in collaboration with the DoE. The DoE distributed the online survey link to schools, with each principal receiving a unique survey link. An accompanying letter of support from the DoE was attached to the consent and survey forms, with the intention of encouraging participation. After 2 weeks, the researcher followed up by email to ascertain each school’s willingness to participate (or to express gratitude for their participation). Each school was encouraged to appoint a person responsible for completing the online survey (principal, vice-principal or representative of the school health team). At every opportunity, including within the study information materials, it was emphasised that participation was voluntary and that school responses would be anonymous and would not be used to evaluate school performance. Data collection were undertaken over 6 weeks from November to December 2022. Data cleaning was performed to verify demographic data and school identity codes (obtained from the MoE database) to ensure that only one response was provided per school.

### Data analysis

The analysis was primarily descriptive with the aim of gaining a broad understanding of what is being implemented in junior schools in Indonesia. ANOVA and Chi-square tests were used to analyse the proportion of different schools that implemented specific policies, categorised by school type (public, private, madrasa). Pearson’s correlation was conducted to clarify associations between variables. Additionally, factor analysis was employed to explore the underlying structure or pattern of the data (for psychometric properties, see Supplement A). Analysis was undertaken using IBM SPSS (Version 29).

## Results

### Participants

At the end of the study period, 161 (42.6%) of 378 schools consented and completed the online survey, which comprised 63 public schools (100% of schools), 74 of 257 private schools (28.8%) and 24 of 56 madrasas (42.8%). Schools varied in their socio-demographic characteristics and other aspects of mental health support. One school reported that 49% of its students experienced mental health problems; it was considered an outlier and was excluded from further analysis.

1  How and to what extent were mental health promotion programs implemented in schools?

**Figure 1:**
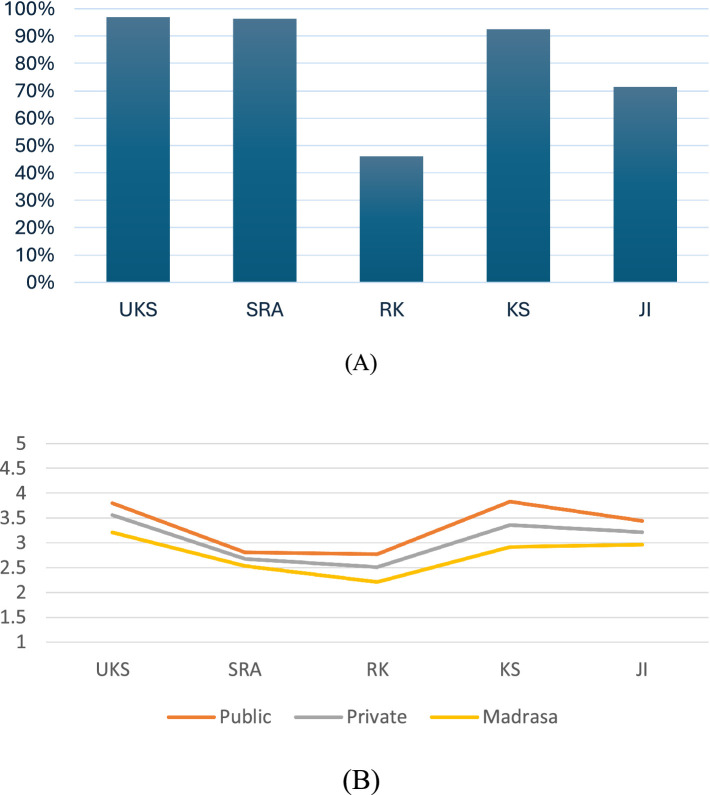
Reported implementation of five school mental health and wellbeing programs. **(A)** Reported implementation by program. **(B)** Perceived implementation success by school type. UKS, School Health Unit (Usaha Kesehatan Sekolah); SRA, Child Friendly School (Sekolah Ramah Anak); RK, Health Education Module (Rapor Kesehatanku); KS, Peer Counsellor (Konseling Sebaya); JI, Mental Health Assessment (Jirona).

Schools reported the implementation of five key mental health-related programs (Table 1). Figure 1 shows the percentage of schools reporting implementation of each of these programs. The highest implementation rates were reported for two of the three national-level policies: the school health unit (UKS) and child-friendly schools (SRA). The peer counsellor (KS) initiative was reported to be implemented well in Surabaya, but implementation was more poorly reported for mental health assessment (JI), a municipality-led policy. The health education module (RK) was implemented the least, despite being a national-level program. Regarding implementation by school type, a lesser proportion of madrasas reported implementing RK, KS and JI than public and private schools.

**Table 1:** Demographic characteristics of schools in Surabaya (*N* = 161).

Variables	Total		Public	Private	MTs
	(*N* = 161)		(n = 63)	(*n* = 74)	(*n* = 24)
	Min–Max	*M* (SD)	*M* (SD)	*M* (SD)	*M* (SD)
Student numbers (N)	10–1,265	491.34 (30.73)	884.45 (29.02)	237.01 (24.45)	206.04 (44.20)
School counsellors (N)	0–9	2.50 (1.60)	3.67 (0.17)	1.84 (0.18)	1.78 (0.23)
School health team (N)	1–7	3.08 (1.72)	3.76 (0.21)	2.81 (0.21)	2.67 (0.37)
External partnerships (N)	0–11	6.94 (3.22)	7.48 (0.40)	6.85 (0.43)	5.89 (0.85)
Prevalence of mental health issues (%)	0–21	3.01 (4.46)	2.03 (0.83)	3.54 (0.55)	3.96 (1.25)

N = Number. M = Mean. SD = Standard deviation. SMP= junior high school. MTs= madrasa on junior school level. Student with problems = Students presenting mental health problems in the past 12 months. External partnerships = The number of external agencies that collaborate around mental health promotion with the school. School health team = The number of members of the school health team who manage mental health issues.

Schools were then asked to report how well they considered they had implemented the program, using a Likert scale question: “Has the school managed to deliver the program?” (1 = no implementation, 2 = well below standard (<50%), 3 = below standard (50–79%), 4 = standard (80–100%), 5 = exceeding the standard). Reasonable success was reported for the implementation of UKS, KS, and JI, with each school type having a mean score above 3 (Figure 1B). Less successful implementation was reported for SRA and RK, and not all schools reported they routinely implemented these two programs.

Public schools reported higher scores on perceived implementation success than private schools and madrasas (details in Supplement B). Specifically, public schools reported higher scores for the perceived implementation success of UKS (*M* (*SD*) _UKSpublic_ = 3.80(0.54), 95% CI = 3.66–3.93) and KS (*M* (SD) _KSpublic_ = 3.83(0.52), 95% CI = 3.69–3.96) than private schools and madrasas. Madrasas reported significantly lower perceived implementation success in all programs (*M* (*SD*) _UKSmadrasa_ = 3.21(0.72), 95% CI = 2.90–3.51; *M* (*SD*) _KSmadrasa_ = 2.92(1.01), 95% CI = 2.49–3.35). No significant differences were found in perceived implementation success by school type for the SRA, RK, and JI programs.

Cluster analysis was performed to advance understanding of differences in reported perceived success. Visual inspection using a dendrogram and cluster comparison suggested two groups: standard and poor-performing schools (see Supplement C). Standardly performing schools were clustered in group 1, reporting standard performance (80–100% perceived success) in implementing UKS, KS, RK, and JI and below-standard performance (perceived success 50–79%) for SRA. Poorly performing schools were clustered in group 2, which reported below-standard performance on all school mental health programs.

2  What are the most important drivers of implementation?

The key drivers we identified around successful implementation of school mental health programs related to scope, school health policies and resources, school and community partnerships, school governance and leadership, and curriculum, as articulated below and in Tables 2–6.

**Table 2:** School policies, resources, leadership and governance approaches to promoting mental health.

No	Approach	Percentage (adj. residuals)					
		Total	Public	Private	MTs	*Χ* ^2^	*Φ*
1	Weekly allocated time to promote mental health (in minutes) Min–Max (0–180), *M* (SD) = 67.96 (45.65)						
2	Scope of actions						
	1. Promoting healthy environments	95	95.3 (0.1)	94.5 (−0.2)	95.8 (0.2)	0.06	0.02
	2. Promoting socio-emotional and spiritual wellbeing	87	93.7 (2)	82.2 (−1.6)	83.3 (−0.6)	4.10	0.16
	3. Mental health as part of comprehensive school health	80.1	84.4 (1)	79.5 (−0.1)	70.8 (−1.2)	1.94	0.11
	4. Preventing problems for all students	72	76.2 (0.9)	67.6 (−1.2)	75 (0.3)	1.37	0.09
	5. Identifying at-risk students	64	65.5 (0.6)	64.4 (−0.1)	58.3 (−0.6)	0.53	0.05
	6. Identifying and target students with psychological problems	72.7	79.4 (1.5)	67.6 (−1.3)	70.8 (−0.2)	2.43	0.12
3	Recipients of school mental health promotion						
	1. Students	51.1	60.3 (1.8)	50 (−0.4)	33.3 (−1.9)	7.71	0.21
	2. Teachers	31.9	39.7 (1.7)	29.7 (−0.5)	17.4 (−1.6)	7.85	0.22
	3. Educational staff	31.3	36.5 (1.2)	29.7 (−0.4)	21.7 (−1.1)	6.57	0.20
	4. Security and sanitation staff	27.5	34.9 (1.7)	25.7 (−0.5)	13 (−1.7)	15.55**	0.31
	5. Parents/families	18.8	15.9 (−0.8)	21.6 (0.9)	17.4 (−0.2)	6.16	0.19
4	Assessment of students						
	1. No assessment	47.2	38.1 (−1.9)	50 (0.7)	62.5 (1.6)	4.58	0.17
	2. Risk factor assessments	76.4	73 (−0.8)	79.7 (0.9)	75.0 (−0.2)	0.88	0.07
	3. Health behaviour assessments	80.7	82.5 (0.5)	82.4 (0.5)	70.8 (−1.3)	1.78	0.10
	4. Learning engagement	83.9	84.1 (0.1)	89.2 (1.7)	66.7 (−2.5)	6.79	0.20
	5. Socio-emotional wellbeing	78.9	77.8 (−0.3)	82.4 (1)	70.8 (−1)	1.53	0.09
	6. Psychological tests	80.1	79.4 (−0.2)	82.4 (0.7)	75 (−0.7)	0.66	0.06
	7. Screening for mental health problems	76.4	73. (−0.8)	81.1 (1.3)	70.8 (−0.7)	1.71	0.0
5	Funding						
	1. No funding	50.9	41.3 (−2)	54.1 (0.7)	66.7 (1.7)	5.02	0.17
	2. Incidental external funding	18	14.3 (−1)	18.9 (0.3)	25.0 (1)	1.42	0.09
	3. Consistent government-only funding	19.9^a^	38.1 (4.6)	8.1 (−3.5)	8.3. (−1.5)	21.57***	0.36
	4. Consistent school budget and multi-source funding	11.2	6.3 (−1.6)	18.9 (2.9)	0.0 (−1.9)	8.95*	0.23
6	School health policies guide implementation and evaluation						
	1. None	31.7^c^	17.5 (−3.1)	39.2 (1.9)	45.8 (1.6)	10.03**	0.25
	2. No written school policies, only best practices	37.9	42.9 (1)	35.1 (−0.7)	33.3 (−0.5)	1.11	0.08
	3. Written policies and clear procedures	30.4	39.7 (2)	25.7 (−1.2)	20.8 (−1.1)	4.38	0.16
7	Reporting school health promotion						
	1. No reporting	32.9	31.7 (−0.3)	31.1 (−0.5)	41.7 (1.0)	0.98	0.07
	2. Government	26.7^a^	46 (4.4)	16.2 (−2.8)	8.3 (−2.2)	20.31*	0.35
	3. School beneficiaries	29.8	1.6 (−6.3)	52.7 (5.9)	33.3 (0.4)	42.65***	0.51
	4. Parent/Community	36.6^b^	30.2 (−1.4)	47.3 (2.6)	20.8 (−1.7)	7.34*	0.21
	5. Others (e.g. PUSKESMAS)	8.7	7.9 (−0.3)	12.2 (0.2)	0.0 (0.1)	0.09	0.02
8	Impact of reporting school mental health promotion						
	1. No impact	41.6	31.7 (−2)	47.3 (1.3)	50 (0.9)	4.20	0.1
	2. Insignificant impact	49.1	55.6 (1.3)	44.6 (−1)	45.8 (−0.3)	1.75	0.10
	3. Significant impact (acknowledgement or rank)	9.3	12.7 (1.2)	8.1 (−0.5)	4.2 (−0.9)	1.73	0.10
9	School health teams exist	83.9 ^a^	96.8 (3.6)	74.3 (−3)	79.2 (−0.7)	13.18***	0.28
	School mental health teams exist						
	1. We do not have a school mental health exists	37.3	47.6 (2.2)	31.1 (−1.5)	29.2 (−0.9)	5.55	0.18
	2. Yes. as a part of the school health team	52.2	41.3 (−2.2)	58.1 (1.4)	62.5 (1.1)		
	3. Yes, as a specifically designated team	10.6	11.1 (0.2)	10.8 (0.1)	8.3 (−0.4)		
10	Members of school health team						
	1. Principal	66.5^a^	84.1 (3.8)	54.1 (−3.1)	58.3 (−0.9)	14.64***	0.30
	2. Teacher	75.8	92.1 (3.9)	66.2 (−2.6)	62.5 (−1.6)	15.09***	0.30
	3. School counsellor	63.4^a^	79.4 (3.4)	58.1 (−1.3)	37.5 (−2.8)	14.74***	0.30
	4. School psychologist	23^a^	27 (1)	21.6 (−0.4)	16.7 (−0.8)	1.18	0.80
	5. Parent representative	26.1	30.2 (0.9)	23 (−0.8)	25 (−0.1)	0.92	0.07
	6. Community	28.6	39.7 (2.5)	21.6 (−1.8)	20.8 (−0.9)	6.26*	0.19
11	Coordinator of the school health team						
	1. We do not have anyone in this role	14.9	7.9 (−2)	20.3 (1.8)	16.7 (0.3)	4.15	0.16
	2. Principal	49.1	61.9	41.9 (−1.7)	37.5 (−1.2)	6.96*	0.20
	3. Vice Principal	4.3	4.8 (2.6)	4.1 (−0.2)	4.2 (0)	0.04	0.01
	4. Teacher	4.3	6.3 (1)	2.7 (−0.9)	4.2 (0)	1.09	0.08
	5. School counsellor	5.6	1.6 (−1.8)	5.4 (−0.1)	16.7 (2.6)	7.49*	0.021
	6. Parent representative	21.1	19 (−0.5)	23 (0.5)	20.8 (0)	0.31	0.04
	7. Community	0.6	0 (−0.8)	1.4 (1.1)	0 (−0.4)	1.18	0.08

*N* = 161. Public= Government funded junior high schools. Private= Community funded junior high schools. MTs= madrasa on junior school level. The scores are percentages of schools’ reported profiles. Adjusted standardized residuals appear in parentheses below group frequencies. Χ2 = Chi-square test was performed on all variables. Φ = Cramer’s V was used to test the effect size of the Chi square. *Post-hoc* comparison was performed using Bonferroni correction. Significant difference found among school types (public, private and madrasa) was indicated with ***p < .001. **p < .01. *p < .05.

a Public schools reported significantly higher than private school and madrasas.

b Private schools reported significantly higher than public schools and madrasas.

c Madrasas reported significantly higher than public and private schools.

**Table 3:** School-community partnerships to promote school mental health.

No	Approach	Percentage (adj. residuals)					
		Total	Public	Private	MTs	*Χ* ^2^	*Φ*
1	School relies on internal case management						
	1. School reported no students with mental health concerns	11.8	4.8 (−2.2)	14.9 (1.1)	20.8 (1.5)	5.54	0.18
	2. School only used external referral (no internal management)	6.8	7.9 (0.4)	6.8 (0)	4.2 (−0.6)	0.39	0.05
	3. School only used internal resources (no external referral)	24.8	12.7 (−2.9)	28 4 (1)	45.8 (2.6)	11.13**	0.26
	4. First, internal case management, and then the external referral is used if further services are needed.	56.5 ^a^	74.6 (3.7)	50 (−1.5)	29.2 (−2.9)	17.48***	0.32
2	School has functional partnerships with external referral systems						
	1. Community health centre at the district level (PUSKESMAS)	33.6	45 (2.5)	26.2 (−1.7))	22.2 (−1.1)	9.7	0.26
	2. Hospitals	25.9	35 (−0.8)	21.5 (−1.1)	11.1 (−1.5)	8.24	0.24
	3. Agency for family violence and neglect cases (PUSPAGA)	18.2	21.7 (0.9)	16.9 (−0.4)	11.1 (−0.8)	11.98	0.29
	4. Agency for addiction cases (BNN)	23.8	31.7 (1.9)	18.5 (−1.4)	16.7 (−0.8)	12.71*	0.29
	5. Agency for juvenile delinquency/minor crimes (KAMTIBMAS)	25.2	33.3 (1.9)	20 (−1.3)	16.7 (−0.9)	7.46	0.22
	6. Health professionals in the community	25.9	30 (1)	24.6 (−0.3)	16.7 (−1)	11.72	0.28
	7. NGO support	14.7	16.7 (0.6)	12.3 (−0.7)	16.7 (0.3)	7.7	0.23
	8. Educational agencies for learning support and strategies	21.7	26.7 (1.2)	20 (−0.4)	11.1 (−1.2)	16.1*	0.33
3	Regular school partners						
	1. Students	51	65 (2.8)	38.5 (−2.7)	50 (−0.1)	10.34*	0.27
	2. Family/parents	39.9	48.3 (1.8)	32.3 (−1.7)	38.9 (−0.1)	3.76	0.16
	3. Other schools	14	10 (−1.2)	15.4 (0.4)	22.1 (1.1)	13.46**	0.30
	4. NGO national level (i.e., WHO Indonesia, UNICEF Indonesia)	6.3	5 (−0.5)	6.2 (−0.1)	11.1 (0.9)	2.55	0.13
	5. NGO local level (i.e., Hotline)	10.5	8.3 (−0.7)	9.2 (−0.4)	22.2 (1.7)	3.34	0.15
	6. Government national level	15.4	20 (1.3)	10.8 (−1.4)	16.7 (0.2)	2.45	0.65
	7. Government local level	25.2^a^	41.7 (3.9)	12.3 (−3.2)	16.7 (−0.9)	18.92***	0.36
	8. Professional Associations (i.e., HIMPSI, IDI)	11.2	8.3 (−0.9)	12.3 (0.4)	16.7 (0.8)	4.62	0.18
	9. Universities	16.1	13.3 (−0.8)	18.5 (0.7)	16.7 (0.1)	7.06	0.22

*N* = 161. Public= Government funded junior high schools. Private= Community funded junior high schools. MTs= madrasa on junior school level. The scores are percentages of schools’ reported profiles. Adjusted standardized residuals appear in parentheses below group frequencies. Χ^2^ = Chi-square test was performed on all variables. Φ = Cramer’s V was used to test the effect size of the Chi square. *Post-hoc* comparison was performed using Bonferroni correction. Significant difference found among school types (public, private and madrasa) was indicated with ***p < .001. **p < .01. *p < .05.

a Public schools reported significantly higher than private school and madrasas.

**Table 4:** The use of school curriculum to promote school mental health (*N* = 161).

No	Approach	Percentage (adj. residuals)					
		Total	Public	Private	MTs	*Χ* ^2^	*Φ*
1	Health issues covered in the last 12 months						
	1. Health behaviours: nutrition, sleep, physical activity and fitness	79.2	85.5 (1.6)	74 (−1.5)	79.2 (0)	9.73	0.24
	2. Substance use prevention	81.4	81 (−0.1)	79.7 (−0.5)	87.5 (0.8)	15.79	0.31
	3. Injury prevention	63.4	69.8 (1.4)	54.1 (−2.3)	75 (1.3)	8.13	0.22
	4. Sexuality and reproductive health	73.9	74.6 (0.2)	68.9 (−1.3)	68.9 (−1.3)	9.85	0.24
	5. Healthy use of technology (e.g., gadgets/internet/social media)	83.9	87.3 (1)	78.4 (−1.7)	91.7 (1.1)	10.20	0.25
	6. Suicide prevention	63.7	65.6 (0.4)	59.7 (−1)	70.8 (0.8)	6.31	0.20
	7. Violence prevention (e.g., bullying, dating violence)	83.1	85.7 (0.7)	79.5 (−1.1)	87.5 (0.8)	8.40	0.23
	8. Stress management (e.g., meditation, stress coping)	67.3	72.6 (1.1)	60.3 (−1.7)	75 (0.9)	7/76	0.22
	9. Improving friendships with peers	84.4	88.9 (1.3)	80.8 (−1.1)	83.3 (−0.2)	7.79	0.22
	10. Improving relationships with parents	84.9	88.7 (1.1)	83.6 (−0.4)	79.2 (−0.9)	12.18	0.27
	11. Improving student relationships with teachers	86.3	90.5 (1.3)	83.6 (−0.9)	83.3 (−0.5)	11.77	0.27
	12. Improving academic self-esteem, motivation and engagement	84.9	88.7 (1.1)	80.8 (−1.3)	87.5 (0.4)	7.28	0.21
	13. Wellness and wellbeing	80.5	84.1 (0.9)	75(−1.6)	87.5 (0.9)	8.97	0.23
2	Curriculum strategies used						
	1. None	12.6	8.1 (−1.4)	16.4 (1.4)	12.5 (0)	2.13	0.11
	2. Core subject	44	50 (1.2)	43.8 (1)	29.2 (−1.6)	3.05	0.13
	3. Carrier subject	67.3	75.8 (1.8)	60.3 (−1.7)	66.7 (−0.1)	3.68	0.15
	4. Infusion subject	60.4	64.5 (0.9)	58.9 (−0.3)	54.2 (−0.7)	0.89	0.07
	5. Co-curricula	67.9	72.6 (1)	74 (1.5)	37.5 (−3.5)	12.03**	0.27
	6. Extra-curricula	63.5	72.6 (1.9)	64.4 (0.2)	37.5 (−2.9)	9.23*	0.24
	7. Psychoeducation	57.9	64.5 (1.4)	58.9 (0.2)	37.5 (−2.2)	5.24	0.18

*N* = 161. Public= Government funded junior high schools. Private= Community funded junior high schools. MTs= madrasa on junior school level. The scores are percentages of schools’ reported profiles. Adjusted standardized residuals appear in parentheses below group frequencies. Χ^2^ = Chi-square test was performed on all variables. Φ = Cramer’s V was used to test the effect size of the Chi square. *Post-hoc* comparison was performed using Bonferroni correction. Significant difference found among school types (public, private and madrasa) was indicated with ***p < .001. **p < .01. *p < .05.

Core health education subject = Skills-based health education can be a core (or separate) subject in the broader school curriculum. Carrier subject = Skills-based health education is sometimes placed in the context of related health and social issues within an existing so-called carrier subject that is relevant to the issues, such as science, civic education, social studies, or population studies. Infusion across multiple subjects = Health topics can be incorporated into all or many existing subjects by regular classroom teachers. Co-curricular activities = Activities that are outside of but usually complement the regular curriculum, such as student newspapers, musical performances, art shows, debates, and competitions. Extra-curricular activities = Activities that develop social skills that are not part of the regular curriculum, such as clubs, engaging in artistic and creative pursuits, volunteering, and participating in community services. Psycho-education activities can introduce health topics as part of information dissemination activities aimed at promoting behavioural changes, such as incidental or regular seminars or student workshops.

**Table 5:** Barriers and enablers of implementation of school mental health promotion (*N* = 161).

Variable	Percentage (adj. residuals)					
	Total	Public	Private	MTs	*Χ* ^2^	*Φ*
Enablers						
1. We have a school health team for mental health promotion	53.4	66.7 (2.7)	48.6 (−1.1)	33.3 (−2.1)	9.01*	0.23
2. Policies and supports that are in place by the government	42.9	50.8 (1.6)	43.2 (0.1)	20.8 (−2.4)	6.37*	0.19
3. The community health centre as a source for mental health assessment and intervention	83.2	90.5 (2)	81.1 (−0.7)	70.8 (−1.8)	5.25	0.18
4. NGOs provide significant help for school mental health promotion	18	19 (0.3)	18.9 (0.3)	12.5 (−0.8)	0.58	0.06
5. Supporting agencies in the community (e.g., PUSPAGA, BNN)	43.5^a^,^b^	58.7 (3.1)	41.9 (−0.4)	8.3 (−3.8)	18.10***	0.33
6. Schools can access teacher training in mental health	21.1	22.2 (0.3)	23 (0.5)	12.5 (−1.1)	1.27	0.09
Barriers						
1. No school health team	16.8	7.9 (−2.4)	23 (1.9)	20.8 (0.6)	5.85	0.19
2. No school mental health team	37.9	28.6 (−2)	44.6 (1.6)	41.7 (0.4)	3.88	0.15
3. No functional relationship with the community health centre	9.3	6.3 (−1)	8.1 (−0.5)	20.8 (2.1)	4.55	0.16
4. UKS is not optimally functional	33.5	23.8 (−2.1)	37.8 (1.1)	45.8 (1.4)	4.99	0.17
5. School lacks mental health literacy to deal with mental health concerns	32.3	25.4 (−1.5)	37.8 (1.4)	33.3 (0.1)	2.42	0.12
6. Lack of access to teacher training in mental health	64	61.9 (−0.4)	66.2 (0.5)	62.5 (−0.2)	0.31	0.04
7. Students are not confident about accessing mental health support services	19.9	14.3 (−1.4)	25.7 (1.7)	16.7 (−0.4)	2.95	0.13
8. Parents’ disapproval of mental health and wellbeing at school	0.6	1.6 (1.2)	0 (−0.9)	0. (−0.4)	1.54	0.09
9. Parents do not support referral services in the community	16.3	11.1 (−1.4)	20.5 (1.3)	16.7 (−0.1)	2.21	0.11
10. Teacher’s poor mental health literacy	18.8	15.9 (−0.8)	23.3 (1.3)	12.5 (−0.9)	1.94	0.11
1. The pandemic changed the school health program	31.7	27 (−1)	37.8 (1.5)	25 (−0.8)	2.43	0.12

*N* = 161. Public= Government funded junior high schools. Private= Community funded junior high schools. MTs= madrasa on junior school level. The scores are percentages of schools’ reported profiles. Adjusted standardized residuals appear in parentheses below group frequencies. Χ^2^ = Chi-square test was performed on all variables. Φ = Cramer’s V was used to test the effect size of the Chi square. *Post-hoc* comparison was performed using Bonferroni correction. Significant difference found among school types (public, private and madrasa) was indicated with ***p < .001. **p < .01. *p < .05.

a Public schools reported significantly higher than private school and madrasas.

b Madrasas reported significantly lower than public and private schools.

**Table 6:** The correlation matrix between the reported implementation of the five key mental health promotion programs in Surabaya with school characteristics.

Variables	UKS	SRA	RK	KS	JI	1	2	3	4	5	6
Total students	0.28**	0.20**	0.16**	0.31**	0.12						
Students with mental health problems	0.13	0.07	0.14	0.13	0.06	0.20**					
School counsellors	0.21**	0.18*	0.15	0.18*	0.20*	0.72**	0.15*				
Members of a school health team	0.28**	0.22**	0.38**	0.37**	0.26**	0.30**	0.13	0.28**			
School external partners	0.15	0.14	0.28**	0.14	0.10	0.16	0.05	0.10	0.26**		
Total perceived enablers	0.23**	0.21**	0.19*	0.30**	0.20**	−0.21*	−0.04	−0.19*	−0.15	−0.30**	
Total perceived barriers	−0.31**	−0.22**	−0.07	−0.26**	−0.08	0.30**	0.22**	0.28**	0.43**	0.30**	−0.14
Enabler—internal resources	0.17*	0.20*	0.16*	0.27**	0.22**						
Enabler—external resources	0.13	0.07	0.09	0.22**	0.14						
Barrier—school’s poor capacity	−0.20*	−0.15	−0.01	−0.11	−0.06						
Barrier—health team’s poor functioning	−0.38**	−0.21**	−0.20**	−0.30**	−0.11						

*N* = 161. UKS, School Health Unit (Usaha Kesehatan Sekolah); SRA, Child Friendly School (Sekolah Ramah Anak); RK, Health Education Module (Rapor Kesehatanku); KS, Peer Counsellor (Konseling Sebaya); JI, Mental Health Assessment (Jirona).

Reported coefficients were analysed using Pearson correlation. **Correlation is significant at the 0.01 level (2-tailed). *Correlation is significant at the 0.05 level (2-tailed). Factor analysis was conducted to examine latent factors for enablers and barriers. Two latent factors for enablers were found: 1) internal resources and access to capacity building, and 2) external resources. Two latent factors for barriers were also found: 1) a school’s poor capacity in mental health promotion and dealing with crisis, and 2) a school health team’s poor capacity.

### Scope

The scope of school mental health promotion strategies refers to the tiered approach promoted by the World Health Organisation (Hendren et al., 1994), with policy focus ranging from promotive strategies for all students, preventive actions for targeted students, to treatment approaches for students with identified concerns (see Table 2). Examples of universal promotive strategies included building a healthy psychosocial environment and strengthening student socioemotional wellbeing. Treatment approaches included assessment strategies to identify students experiencing psychological problems.

### School health policies and resources

Most participating schools made some effort to map their current profile of school mental health promotion programs (see Table 2). On average, schools reported allocating approximately one hour per week of dedicated time to mental health promotion, encompassing both in-classroom and out-of-classroom activities. Schools routinely targeted students (rather than families or teachers) as their primary recipients. Nevertheless, only about one-third of madrasas reported regularly supporting their students’ emotional wellbeing. Schools employed multiple assessment strategies to gather information about their students’ mental health, wellbeing, and learning engagement.

Approximately one-third (30.4%) of schools reported that they had neither evaluated nor reported their school mental health programs within the last 12 months. Most schools reported that there was little or no impact of reporting or not reporting (e.g., little or no incentive). As for funding, half reported no reliable funding for mental health promotion. Public schools heavily relied on government funding to support program implementation. Most madrasas did not commit any funding for school-based mental health promotion programs.

### School governance and leadership

Most schools reported having a school health team to address general health issues, consistent with recommendations in the UKS policy. Despite this, only 10.6% reported having a designated school mental health team (Table 2). The mental health team was typically reported to be part of the general school health team, consisting of teachers, school leaders, and counsellors. Most school health teams were led by principals. Some teams were also reportedly led by parents, particularly in private schools. About 15% of schools reported not having a coordinator for their school health team.

### School and community partnerships

Schools reported both internal and external case management strategies. Most schools reported combining internal and external strategies for case management; however, differences were observed in the reported use of internal strategies (Table 3). Some schools reported they relied heavily on external referrals due to limited internal capacity. In contrast, other schools, particularly madrasas, reported relying on internal case management rather than strategies involving external partners. This is in the context that about one in 10 schools reported not having any students with mental health problems in their schools.

Just over a third (33.6%) of schools had a relationship with their local community health centre (Puskesmas), which is intended to function as the primary referral service for schools. It should be noted that some schools reported to have incidental or non-functional partnerships with Puskesmas (57%), and the rest reported no interaction. Some public schools used wider external services (e.g., General Practitioners, psychologists, other specialists in the community). Most schools had a limited number of functional partnerships with local NGOs and government supporting agencies in the community (e.g., Puspaga for family violence, BNN for addiction problems).

### School curriculum

Most public schools used carrier subjects such as science to embed aspects of health education. Some schools also chose to infuse health topics into regular academic subjects (e.g., social science, languages) and disseminated health components in co-curricular and extra-curricular health education (see Table 4). Many health issues were covered in schools, from enhancing protective relationships (e.g., between students with peers, parents, and teachers) to specific areas of mental health (e.g., stress management, substance abuse and violence prevention).

1. What are the enablers and barriers to implementation?

Perceived barriers and enablers of school mental health promotion were sought to identify critical targets that could be leveraged to support implementation (see Table 5). In terms of enablers, most schools (83.2%) viewed their local community health service (Puskesmas) as a resource for mental health assessment and intervention. School health teams were also viewed as enablers by over half the schools (53.4%). While community supports were also strongly endorsed (43.5%), there were significant differences by school type, with madrasas appearing to have fewer community linkages. A minority of schools (21.1%) reported access to teacher training around mental health, which was also less endorsed by madrasas. In general, enablers were more endorsed in public schools than in private schools and were least endorsed by madrasas.

Regarding barriers, schools reported lack of access to teacher training in mental health (64.0%) and poor capability of the school mental health teams to implement and monitor mental health promotion. Poor mental health literacy was endorsed as a barrier to school mental health promotion (32.3%). This likely reflects poor community mental health literacy given the proportion of schools that endorsed poor mental health literacy among teachers (18.8%); parents who did not support referrals to services in the community (16.3%); and students who lacked confidence in accessing mental health support services (19.9%). No functional relationship with the community health centre was reported by 9.3% of respondents.

A correlational analysis was performed to profile the contributing factors to perceived implementation success (see Table 6). This revealed that higher numbers of enablers and fewer barriers were associated with greater perceived implementation success. Factor analysis further explored the nature of barriers and enablers. Two latent factors were identified within the enablers (internal enablers and external enablers) and within the barriers (the school’s poor implementation capacity and the school health team’s poor functioning) (see Supplement D). Correlation using latent factors revealed that schools’ perceived success was higher when they reported having stronger internal resources (e.g., a functional school health team and easier access to teacher training) and greater capability to respond to health issues in schools (e.g., a strong school health team and partnership with their local community health service).

In addition to these correlations with the single item relating to perceived implementation success, a Chi-square test of independence revealed a positive association between perceived implementation success with having a school health team that can work on promoting mental health, *Χ*^2^(1, *N* = 161) = 6.91, *p* = 0.009. *Φ* = 0.20. In line with this, less than optimal implementation was also more likely to have occurred when a school had a non-functional school health unit, *Χ*^2^(1, *N* = 161) = 7.39, *p* = 0.001. *Φ* = −0.21. Further triangulation from other variables (e.g., barriers and enablers) is shown in Supplement C; Table 1.

1. What is needed to improve school mental health promotion?

In general, schools reported needing more support for teacher training in mental health promotion (88.2%), building mental health literacy among teachers (80.1%), funding (75.8%), and enhanced school leadership (58.4%) (Table 7). There was also recognition of the importance of regular mental health literacy training for students (83.9%) and of support from parents and guardians (79.5%). There was less variation by school type, although madrasas endorsed these items to a lesser extent. The most commonly reported roles for schools to enhance school mental health promotion were to build students’ mental health literacy (81.4%), supported by policies and guidelines around mental health (80.7%), school health teams (68.3%) and opportunities to refer individual students with possible mental health needs for assessment (67.7%). There was less variation by school type in this regard, although madrasas reported the lowest scores on all perceived roles.

**Table 7:** Support and roles needed to improve school mental health promotion (*N* = 161).

Improving school mental health promotion	Percentage (adj. residuals)					
	Total	Public	Private	MTs	*Χ* ^2^	*Φ*
Support needed to improve mental health promotion						
1. Funding	75.8	76.2 (0.1)	77 (0.3)	70.8 (−0.6)	0.38	0.05
2. Regular teacher mental health promotion training	88.2	92.1 (1.2)	87.8 (−0.1)	79.2 (−1.5)	2.79	0.13
3. Regular student mental health training	83.9	88.9 (1.4)	82.4 (−0.5)	75 (−1.3)	2.67	0.13
4. Greater school leadership capacity	58.4	58.7 (0.1)	60.8 (0.6)	50 (−0.9)	0.87	0.07
5. Greater mental health literacy of teachers	80.1	81 (0.2)	81.1 (0.3)	75 (−0.7)	0.46	0.05
6. Community linkages	65.8	69.8 (0.9)	67.6 (0.4)	50 (−1.8)	3.22	0.14
7. Referral pathways	72.7	74.6 (0.4)	74.3 (0.4)	62.5 (−1.2)	1.47	0.09
8. Stronger understanding and support from family/parents	79.5	77.8 (−0.4)	83.8 (1.2)	70.8 (−1.1)	2.05	0.11
School roles to promote mental health						
1. Establish a School Health Team	68.3	71.4 (0.7)	68.9 (0.1)	58.3 (−1.1)	1.4	0.09
2. Make policies and guidelines around mental health	80.7	81 (0.1)	82.4 (0.5)	75 (−0.8)	0.61	0.06
3. Support mental health promotion at school	62.7	68.3 (1.2)	62.2 (−0.1)	50 (−1.4)	2.49	0.12
4. Profile student mental health needs	43.5	46 (0.5)	48.6 (1.2)	20.8 (−2.4)	5.98	0.19
5. Profile school mental health needs and current resources	46.6	42.9 (−0.8)	54.1 (1.8)	33.3 (−1.4)	3.70	0.15
6. Improve teachers’ mental health literacy	74.5	79.4 (1.1)	78.4 (1)	50 (−3)	8.96*	0.23
7. Improve parents’/carers’ mental health literacy	62.7	66.7 (0.8)	63.5 (0.2)	50 (−1.4)	2.1	0.11
8. Improve students’ mental health literacy	81.4	77.8 (−0.9)	89.2 (2.4)	66.7 (−2)	6.94*	0.20
9. Engage with policymakers and community stakeholders	46	50.8 (1)	43.2 (−0.6)	41.7 (−0.5)	0.99	0.07
10. Implement relevant curriculum around mental health promotion	44.1	44.4 (0.1)	48.6 (1.1)	29.1 (−1.6)	2.79	0.13
11. Refer students with possible mental health issues for assessment	67.7	74.6 (1.5)	70.3 (0.6)	41.7(−3)	9.03*	0.23
12. Refer students to health professionals and services in the community	54	60.3 (1.3)	56.8 (0.6)	29.2 (−2.7)	7.18*	0.21
13. Provide learning support for students with mental health needs	54.7	58.7 (0.8)	52.7 (−0.5)	50 (−0.5)	0.74	0.06

*N* = 161. Public= Government funded junior high schools. Private= Community funded junior high schools. MTs= madrasa on junior school level. The scores are percentages of schools’ reported profiles. Adjusted standardized residuals appear in parentheses below group frequencies. Χ^2^ = Chi-square test was performed on all variables. Φ = Cramer’s V was used to test the effect size of the Chi square. *Post-hoc* comparison was performed using Bonferroni correction. Significant difference found among school types (public, private and madrasa) was indicated with ***= *p* < .001. **= *p* < .01. *= *p* < .05.

## Discussion

This study found that junior high schools in Surabaya were markedly diverse in their reported capacity to implement school mental health promotion, with variation by school type (public, private, madrasa), capacity, and resources. Regardless of these differences, most schools, including madrasas, attempted to implement various school mental health promotion programs as mandated by school health policies at national and local levels.

Complex and multi-faceted tasks of school mental health would ideally require a designated school mental health team that is skilled at delivering both universal and targeted programs using a sustainable and integrated strategy that includes attending to school leadership, regular staff training and access to responsive health services, both within schools and the community (Rones and Hoagwood, 2000; Kern et al., 2017; Richter et al., 2022). In reality, most schools in Surabaya appeared to be struggling to form a designated school mental health team with an appropriate governance model, which may reflect a lack of skilled personnel and poor mental health literacy. Additionally, schools reported being constrained by poor leadership, lack of funding for mental health promotion, inadequate referral systems, and limited external partnerships, which will further limit the sustainability of any actions designed to enhance mental health. The quality of school mental health services could not be determined due to the lack of school-level policies to specifically guide internal case management and external referrals, as well as inadequate guidance on monitoring, evaluation, and reporting of school mental health programs. These findings suggest that schools in Surabaya face multiple challenges including inadequate monitoring strategies, funding, and policies for monitoring and evaluation, which are likely to result in less-than-optimal implementation.

The reported implementation success of all five school mental health programs identified in our prior qualitative study (Margaretha et al., under review, see footnote 1) ranged from ‘standard’ to ‘poor’ performance. Beyond the lack of capacity around mental health promotion, this may also reflect competing priorities, as schools in Indonesia continue to struggle to improve the quality of their education systems to achieve optimal educational targets (Sukmayadi and Yahya, 2020). This situation is similar to research findings in other LMICs, as while school mental health is reportedly one of many health priorities, challenges are consistently reported around implementation (Brooks et al., 2019; Kucera et al., 2023).

We found that public schools generally reported higher scores of perceived implementation success than private schools and madrasas. This performance gap may indicate a significant underlying governance problem. Our previous work showed that public schools, under the direct management of the MoE and DoE, were more likely to be prioritised with assistance and support (i.e., routine government funding, teacher training) than private schools and madrasas, which would be expected to result in them being better equipped to fulfil their role in promoting mental health. In Indonesia, madrasas are governed by the MoR and DoR, which may result in a disconnection from the school health policies that are largely driven by the MoH and managed through the MoE and DoE. This difference appears to result in substantially poorer capacity to implement school health policies (i.e., the schools that reported below-standard performance on all school mental health programs). An immediate priority to address this apparent inequity would be to target schools with more limited resources, most notably madrasas and poorly-resourced private schools. Given the importance of school linkages to community health services, the MoH could potentially provide assistance to monitor the implementation of school mental health promotion delivered in schools under the MoE and in madrasas under the MoR. Those schools identified as having poorer capacity to implement school mental health policies could then be supported by specific engagement from the DoE and the DoR. Our findings suggest that this would need to include opportunities for teacher training and access to school counsellors. Overall, these findings underline the urgency of accelerating capacity building in school mental health promotion in Surabaya. In the future, more explicit collaboration between the MoH, MoE and MoR around the development, implementation and monitoring of school mental health promotion may achieve better school engagement, commitment and participation.

Notwithstanding the broad benefits of engaging families in schools, parent commitment to academic success at all costs can potentially hinder the impact of school mental health promotion. For example, parental involvement focused on promoting education and academic achievement generally guides students to reach optimal mental health, but not for those who are experiencing cyber-bullying, for whom parental involvement can become an extra burden (Wang et al., 2019). Additionally, parent involvement naturally evolves over time. As adolescents grow older and seek more independence from their parents, parents who are overinvolved in schooling or overly controlling can be negatively perceived by students. The current study found that parent leadership (rather than participation) could undermine the value of schools developing more professionally-led approaches. For example, some schools reported that parents served as coordinators of the school health team. Beyond confidentiality concerns for individual students and families, this risks undermining the value of professional mental health personnel on school health teams. While schools recognised the value of parents being supportive of school mental health promotion, as well as approaches to build parent mental health literacy and their support of community referrals, it is also important that their roles are appropriate for the task (Comer and Haynes, 1991). Parental involvements can be oriented toward supporting students’ mental health through volunteering, communication with teachers/school counsellors and attending school meetings (Comer and Haynes, 1991; George et al., 2014; Profe and Wild, 2017; Wang et al., 2014).

We identified two implementation strengths. *Firstly*, it appeared that both top-down (national) and bottom-up (local) policies were the drivers of school-based mental health promotion programs. The socialisation and implementation of top-down school health policies (UKS, RK, and SRA) may help schools appreciate their role as the enabling context, which could incentivise bottom-up initiatives (KS and JI) from schools, communities, and local governments. Given that some schools have developed their own programs, it is apparent that the function of schools and local governments extends beyond merely executing national mandates to actively addressing mental health challenges in their local contexts. This finding is relevant for other LMICs, as it suggests that governments that promote school mental health through national policies and mandates can provide a context for schools to proactively respond to their local conditions. *Secondly*, most schools reported addressing multiple health topics within their school health teams, indicating familiarity with the comprehensive school health framework of health-promoting schools (Margaretha et al., 2023, see footnote 1). This aligns well with the function of UKS as an umbrella for various programs targeting different health issues, including mental health promotion. For example, previous studies have reported that mental health assessments, such as JI, complemented the UKS program in schools in Surabaya. In these studies, JI assessment was used to identify students at risk of developing mental health disorders or substance abuse, with the results used to plan intervention strategies through UKS services or referrals of individual students to community health services (Kholifah et al., 2022; Wibrata et al., 2022).

In this study, schools reported a spectrum of mental health interventions, ranging from universal promotion and selective prevention to indicated prevention and early intervention, and treatment of disorders of varying severity. Recognizing the wide range of interventions that can be implemented in schools, the question arises as to which strategies should be prioritized for mental health interventions in schools. Different conceptualisations of mental health can influence considerations of priority strategies. For example, those coming from the perspective of positive mental health are likely to prioritize promotive actions, whilst those with more clinical perspectives may prioritize strategies for preventing and treating mental health disorders. Positive conceptualizations of mental health and promotive approaches are likely to sit well with the notion of schools as centers of learning and studies show that positive approaches to mental health are more effective in schools (Greenberg et al., 2000; Jennings and Greenberg, 2009). Yet schools also need students to be able to access appropriate treatment services.

Synthesising these research findings suggests that the Indonesian government could commit to three important investments with the goal of creating an enabling context for school mental health promotion. *Firstly*, the national government could establish a higher-level policy regarding minimum standards for school mental health promotion, including access to quality clinical services within schools and clear referral pathways beyond schools. Currently, standardised policies regarding school mental health promotion are scarce, with only one manual available for school mental health services in Indonesia (Ministry of Health Republic of Indonesia, 2018). While the inclusion of mental health within general school health policies provides a positive policy context for schools, the lack of specific guidance for school mental health and wellbeing will impose a significant obstacle to future progress. The development of standards for school mental health promotion would ideally regulate the provision of routine funding, facilities, and infrastructure, as well as human resources and training, and outline the forms of mental health services that should be provided in schools (e.g., internal case management).

*Secondly*, investment in school leadership is warranted to ensure that school leaders can support their staff in gaining an appropriate level of mental health literacy and management capacity to deliver health programs that support both mental health and academic outcomes. To do so, schools must understand their roles and invest in building their capacity. A crucial step for schools is to equip staff with the necessary knowledge and skills to effectively manage the school mental health team.

*Thirdly*, the DoE must deliberately strengthen schools’ implementation capabilities which would be aided by collaborations and partnerships with various stakeholders (i.e., DoH, DoR, government supporting agencies, universities, professional associations, NGOs, and the community). For example, the DoE can only develop internal case management and external referral systems when it partners with the DoH (the DoH is responsible for community-based health services). In essence, schools need to be supported in developing their capabilities to promote mental health and well-being through the establishment of school-level policies, the facilitation of curriculum and education, familiarity with referral and healthcare systems, and the provision of services and support for students, staff, and parents (O’Reilly et al., 2018; Brooks et al., 2019). In performing this task, the DoE needs to involve the DoR to ensure that the authority over religious schools has the commitment to implement mental health promotion and the capacity to partner, including with community health centres.

This study has several limitations. *Firstly*, the rating of school implementation success was largely assessed by one item, which is subject to subjective bias and social desirability effects. However, as this study was part of an exploratory mixed-method design where the measurement tool was developed from previous qualitative findings (Margaretha et al., under review, see footnote 1), the results of this large survey can be compared to our qualitative findings, including those related to implementation success. It was reassuring that the same pattern of findings regarding implementation was apparent from the qualitative study, namely, better practices in public schools and poorer practices in madrasas. In addition, perceived implementation success was supported by data triangulation from enablers and barriers; schools were more likely to report standard performance (rather than poor performance) when they had a school mental health team and a functional school health unit that supported school mental health promotion.

*Secondly*, in comparison to the high participation rate in public schools, the lower participation rate in private schools and madrasas could lead to selective reporting bias. As the DoE led the communication with schools, this disproportionate participation may reflect differences in communication between the DoE and private schools, as well as between the DoR and madrasas. It may also reflect lower levels of perceived importance from those schools due to differences in governance. Finally, it could reflect lower interest or less understanding of school mental health promotion among the non-participating schools and madrasas. These differences in response rates suggest that comparisons between school types should be interpreted cautiously and considered more as hypothesis-generating than confirming.

*Thirdly*, the collected data were descriptive and was not intended to be used for making causal analyses and conclusions. This research was also not intended to function as an evaluation of program implementation in schools. Despite these limitations, the assessment approach developed in this research has the potential to be further developed to map the implementation of school mental health programs, especially if accompanied by opportunities for objective confirmation of school self-reported practices.

To strengthen our understanding about the actual implementation of school mental health promotion programs in Indonesia, future research would need to address the above limitations. For example, using multiple-item instruments with sound psychometric properties to measure implementation effectiveness, and applying such measurements in a representative sample consisting of all school types. A collaboration between the MoE and the MoR to undertake action research could be expected to optimize participation from public schools, private schools, and madrasas.

## Conclusion

These findings suggest that junior high schools and madrasas in Surabaya appreciate that mental health promotion lies within their school health programs, and that most schools are engaged in multiple programs related to mental health and wellbeing. The implementation of school mental health promotion in Indonesia was driven by national government policies (top-down policies) but was also supported by local government and community initiatives (bottom-up policies). Arguably, a strength of school mental health promotion in Indonesia is that the implementation of programs appears to be based on a comprehensive school health framework, in which mental health promotion is delivered as part of the wider provision of health within the UKS framework.

To leverage schools’ mental health capacity in the future, schools will need to strengthen their various roles, with greater need for planning and investing in building their capacity to implement mental health promotion, including preparing skilled personnel to function within school health teams. At the same time, governments will need to optimise their role in building the enabling context for school mental health promotion through investing in whole-school strategies. Greater orientation to equity is urged, appreciating the apparent variation in capacity of individual schools. These findings also underscore the importance of collaboration among government agencies and schools to accelerate the implementation of school mental health promotion in Surabaya. In summary, the Indonesian government has a significant opportunity to advance school mental health.

## Data Availability

The original contributions presented in the study are included in the article/Supplementary material. Further inquiries can be directed to the corresponding author.

## Footnotes

1 Margaretha, M., Azzopardi, P. S., Fisher, J., and Sawyer, S. M. (under review). School mental health in Indonesia: challenges and opportunities.
