# Supplementary material for: School mental health promotion in Indonesia: a quantitative survey from Surabaya
Source: Front Psychol. 2026 Feb 16;16:1680302. doi: 10.3389/fpsyg.2025.1680302 (PMC12950719; doi:10.3389/fpsyg.2025.1680302)
Supplement: Supplementary file 1 [file Data_Sheet_1.docx]

**Supplement A. Blueprint of the school survey**

Table 1. Blueprint of the school survey

| **Component** | **Dimension** | **Operationalisation** | **Information of interest** | **Response** | | **Psychometric properties** | |
| --- | --- | --- | --- | --- | --- | --- | --- |
| **Part 1. Mental health framework in schools (n= 25 items)** | | | | |  | |  |
| **Scope** | *Multi-tiered school mental health model* | The percentage of schools that have implemented a multi-tiered model in school mental health, from the promotion, prevention of mental health problems, and treatment/support for students with clinical diagnoses. | *Has your school conducted any of these approaches in the last 12 months?*   - 1. We identify and target students with significant mental health problems.   2. We identify and target students at higher risk of developing mental health problems.   3. We run programs for all students that aim to prevent mental health problems.   4. We do not address mental health separately but as part of a comprehensive health approach   5. We provide emotional and spiritual wellbeing programs   6. We aim to create healthy physical and psychosocial environments   7. Other, please specify… | Please choose one response for each item:  0= No  1= Yes | | Reliability *α* = .92  Exploratory Factor analysis found 3 latent factors (promotive, preventive, treatment) with explained variance 71.70%. | |
| **Approach** | *Government school health policies* | The percentage of schools that have implemented the mandated school mental health policies. | *Is your school currently implementing any of the following* ***mental health programs****?*   1. Usaha Kesehatan Sekolah 2. Sekolah Ramah Anak 3. Rapor Kesehatanku 4. Konselor Sebaya 5. Assessment Jiwa Rokok Narkoba (JIRONA) 6. Other, please specify… | Please select all that apply (you can choose more than one).  0= No  1= Yes | | N/A | |
|  |  |  | If so, has your school managed to deliver this (x) program in accordance with the expected implementation standards? | Please select one that applies  1= no, it is far below the standard (<50%)  2= no, it is below the standard (51-79%)  3= yes, it is up to the standard (80-100%)  4= yes, it is exceeding the standard (> 100%) | | N/A | |
|  |  |  | If so, will you continue this (x) program? | 0= No  1= Yes | |  | |
|  | *Profiling school policies and resources* | School has identified resources (e.g. time allocation, data collection strategy) and needs (e.g. targeted recipients, funding, school needs) for mental health promotion. | *How many hours per week does the school health team commit/allocate for promoting student mental health and wellbeing?*  *Who have been the recipients of any school mental health promotion in the past 12 months?*   1. Students 2. Teachers 3. Educational staff 4. Security and sanitation staff 5. Parents/families 6. Other, please specify…. | Please give number ….. in minutes.  Please choose one response for each item:  0= never  1= incidentally  2= regularly | | N/A  N/A | |
|  |  |  |  |  |  |  |  |
|  |  |  | *Please indicate whether your school has conducted any of the following assessments in the past 12 months?*   1. We do not conduct any assessments 2. Risk factor assessments (e.g. tobacco use, drug use) 3. Health behaviour assessments (e.g. sleeping, nutrition, physical activity and inactivity) 4. Learning engagement and wellbeing (e.g. motivation, perseverance, academic self-esteem) 5. Psychological tests (such as personality, aptitude, interest) 6. Screening for mental health problems (such as suicidal behaviours, hyperactivity, emotional problems) 7. Other, please specify…. | Please select all that apply (you can choose more than one).  Please indicate:  1= for students  2= for school staff and teachers  3= for families | | Reliability for student *α* = .88  Exploratory Factor analysis found 1 latent factor with explained variance 64.75%. | |
|  |  |  | *Has the school secured source(s) for funding school mental health promotion throughout the year?* | Please select one that applies.  0= No, we do not have funding  1= Yes, sufficient, but only comes from external grants. We have only as an incidental program, as we have not included this in our school's annual budget.  3= Yes, there is a consistent source for the whole-year program only from the government funding (it is included in our annual school budget).  4= Yes, there are consistent sources for the whole-year program from the government, community and annual school funding. | | N/A | |
|  | *Monitoring, and evaluation*  Reference*:*  FRESH monitoring and evaluation (UNESCO, 2013) | School has identified monitoring and evaluation strategies. | *Are there procedures in place to monitor and enforce the school health and socio-emotional wellbeing policy at the school level?* | Please select one that applies.  0 = There is no school health policy document, or no procedures in place to monitor and enforce.  1 = There are plans to establish school-level procedures to ensure the school health policy is followed.  2 = Policy documents and clear procedures are established to ensure that the school health policy is followed and monitored. | | N/A | |
|  |  |  | *To whom has the school reported any school health reports in the last 12 months?* | Please select one that applies.  0= School does not report  1= Government  2= School beneficiaries  3= Parent/community  4= Others | | N/A | |
|  |  |  | *Are there any consequences or responses for the school following the submission of reports on mental health promotion?* | Please choose one that applies:  0= No  1= Yes, but it is insignificant (e.g. only as an internal evaluation system).  2= Yes, and it is significant (e.g. receiving acknowledgement or rank as an achievement from the government or external agencies). | | N/A | |
|  | *School governance and leadership* | The school has identified leadership strategies (e.g. school health team, school mental health team) for conducting mental health promotion. | Does your school have a school health team?  Does your school have a school mental health team?  *Who is on this school health team?*   1. We do not have a school health team 2. Principal 3. Teacher 4. School counsellor 5. School psychologist 6. Parent representative 7. Community 8. Other, please specify   Who is responsible for planning, coordinating, and evaluating the school's approach to promoting mental health?   1. We do not have anyone in this role 2. School health coordinator 3. Principal 4. Teacher 5. School counsellor 6. Parent representative 7. Community 8. Other, please specify… | Please select one that applies  0= No  1= Yes  Please select one that applies  0= No  1= Yes, they work as a part of the school health team (the school health team also targets mental health promotion for the whole school in their current work)  2= Yes, there is a separate unit from the school health team (focus only on promoting school mental health and wellbeing)  Please select all that apply. You can choose more than one answer (unless you choose 1).  Please select one that applies. | | N/A  N/A  N/A  N/A | |
|  |  |  |  |  |  |  |  |
|  |  |  |  |  |  |  |  |
|  |  |  |  |  |  |  |  |
|  | *Effective partnerships between schools and communities.* | School has familiarised itself with internal and external referral and functional partnerships in facilitating school mental health promotion. | *How does your school deal with any students with mental health concerns within the school (internal)?*  1= We never have any students with mental health concerns  2= We never handle any student cases within the school. We directly refer the case to professionals in the community (external).  3= We use our internal resources to work with students with mental health concerns (no external referral).  4= We first try to handle cases in the school, starting with an internal referral where students can access services from school staff (eg school counsellors/psychologists). However, if further services are needed, we will refer cases to professionals in the community.  5= Other, please specify… | *Please select an answer that describes the condition of your school.* | | N/A | |
|  |  |  | *Which of the following does your school refer to (externally) once you have identified a student with mental health concerns?*   1. Refer to GP in PUSKESMAS – PKPR (Penyuluhan Pelayanan Kesehatan Peduli Remaja) 2. Refer to the hospital 3. Refer to PUSPAGA (Pusat Pembelajaran Keluarga) *for family violence and neglect cases 4. Refer to BNN (Badan Narkotika Nasional) *for addiction cases 5. Refer to KAMTIBMAS (Keamanan dan Ketertiban Masyarakat) *for juvenile delinquency/minor crimes 6. Refer to specialist health professionals in the community (e.g. psychologists, pediatrician, psychiatrist) 7. Request support from NGO (e.g. Hotline Surabaya) 8. Refer to educational agencies for learning support and strategies (e.g. individual education plan) 9. Other, please specify… | Please choose one response for each item:  0= Never, as most teachers do not know about this system  1= Incidentally, but our school is not yet familiar with how this system operates.  2= Incidentally, with some teachers (e.g. only school counsellor) are familiar with how this system operates.  3= We know we can access this system whenever we need support. Most teachers are familiar with how this system operates, as our school has established a functional network with it. | | Reliability *α* = .94  Exploratory Factor analysis found 1 latent factor with explained variance 73.20%. | |
|  |  |  | *What partners does the school collaborate with to promote mental health and wellbeing?*   1. Students 2. Family/parents 3. Other schools 4. NGO national level 5. NGO local level (i.e. LSM Hotline) 6. Government national level (i.e. WHO Indonesia, UNICEF Indonesia) 7. Government local level (i.e. hotline) 8. Governmental agencies (i.e. BNN, PUSPAGA, PUSKESMAS - PKPR) 9. Professional Associations (i.e. HIMPSI, IDI) 10. Universities 11. Other, please specify… | Please choose one response for each item:  0= We do not have this partnership  1= Incidental partnership 2= Regular partnership throughout the year | | Reliability *α* = .89  Exploratory Factor analysis found 1 latent factor with explained variance 51.53%. | |
|  | *School socio-emotional environment*  Reference:  Safe environment from PSE profile (WHO, 2003) | The percentage of schools that meet the national physical and socio-emotional safety standards. | *Has your school undertaken any of the following* ***specific actions*** *in the last 12 months that aim to create a* ***healthy psychosocial environment****?*   1. Actions to provide a friendly, rewarding and supportive atmosphere 2. Actions to support cooperation between students 3. Actions to encourage active learning 4. Actions to prevent bullying and harassment between students 5. Actions to prevent physical punishment and violence by teachers 6. Actions to promote the availability of creative activities 7. Actions to encourage connections with nature 8. Actions to connect school and home life 9. Actions to promote equal opportunities for boys and girls 10. Other, please specify… | Please choose one response for each item:  0= No  1= Yes | | Reliability *α* = .94  Exploratory Factor analysis found 1 latent factor with explained variance 65.22%. | |
|  | *School health education/curriculum*  Reference**: S**kills-based health education (WHO, 2003) | The percentage of schools that provide regular skills-based health education sessions, as recommended in the national guidance. | *As part of* ***health education****, has your school addressed any of the following issues in the last 12 months?*   1. Healthy behaviour: nutrition, sleep, physical activity and fitness 2. Substance use prevention (tobacco, alcohol, marijuana, heroin, amphetamine, etc.) 3. Injury prevention 4. Sexuality and reproductive health (including puberty, gender identity, pornography, HIV/AIDS) 5. Healthy use of technology (e.g., gadgets/internet/social media) 6. Suicide prevention 7. Violence prevention (e.g., bullying, dating violence) 8. Stress management (e.g., meditation, stress coping) 9. Improving friendships with peers 10. Improving relationships with parents 11. Improving student relationships with teachers 12. Improving academic self-esteem, study motivation and learning engagement 13. Wellness and wellbeing 14. Other, please specify…. | Please specify grade(s):   1. None 2. Yes, in year 7 3. Yes, in year 8 4. Yes, in year *9* 5. Yes, in multiple years | | Reliability *α* = .95  Exploratory Factor analysis found 1 latent factor with explained variance 65.30%. | |
|  |  |  | For the majority of the topics, how are they taught?   1. We do not apply this in health education 2. A core health education subject – Skills-based health education can be a core (or separate) subject in the broader school curriculum. 3. Carrier subject – Skills-based health education is sometimes placed in the context of related health and social issues within an existing, so-called carrier subject that is relevant to the issues, such as science, civic education, social studies, or population studies. 4. Infusion across many subjects – Health topics can be included in all or many existing subjects by regular classroom teachers. 5. Co-curricular activities – activities that are outside of but usually complement the regular curriculum, such as: student newspapers, musical performances, art shows, debate, and competitions. 6. Extra-curricular activities – activities for developing social skills but not related to the normal curriculum, such as joining clubs, artistic and creative pursuits, volunteering, and community services. 7. Psycho-education activities such as incidental or regular seminars or student workshops. 8. Other, please specify… | Please select all that apply (you can choose more than one). | | N/A | |
|  | *School physical environment* | School demographics | *My school is -* **type**  Public school  Private school  Private school based on religious values  Madrasah Tsanawiyah | Please select one that applies. | | N/A | |
|  |  |  | *The population in my school includes*   1. Student presenting mental health problem in the past 12 months (girl): 2. Student presenting mental health problem in the past 12 months (boy): 3. School counsellor: 4. Student body: 5. Total whole-school population: | Please answer in numbers (frequency). | | N/A | |
|  | *Enablers and barriers* | The percentage of schools that reported enablers of implementation. | *Which of the following enablers have supported your school in implementing mental health promotion?*   1. We have a school health team that manages mental health promotion approaches 2. We are aware of the policies and supports that are in place by the government, which are sufficient guidelines for implementation 3. PUSKESMAS serves as a source for mental health assessment and intervention 4. NGOs provide significant help for school mental health promotion 5. We can locate other sources of support in the community (e.g. BNN, universitas, professional association) 6. We can access teacher training in mental health 7. We have accessed mental health training for teachers from the Education Department of Surabaya 8. Other, please specify… | Please select all that apply - you can choose more than one answer. | | Reliability *α* = .67  Exploratory Factor analysis found 2 latent factors (internal enablers and external enablers) with explained variance 51.85%. | |
|  |  | The percentage of schools that reported barriers to implementation. | *Which of the following barriers has your school been dealing with that prevents it from implementing mental health promotion?*   1. We do not have a school health team 2. We do not have a school mental health team 3. We do not have a functional relationship with PUSKESMAS 4. Our school UKS is not optimally functional 5. Our school does not have sufficient mental health literacy to deal with mental health concerns 6. We lack access to teacher training in mental health 7. Our students are not confident in accessing support services at school for mental health issues (e.g. anxiety) 8. We do not have sufficient funding to implement the activities we would like to offer 9. Parents disapprove of any focus on mental health and wellbeing by schools 10. Parents do not support referrals of students with mental health concerns to support services at school 11. Parents do not support referrals of students with mental health concerns to support services in the community 12. The pandemic has caused changes in our school health program, so we can no longer focus on mental health 13. Other, please specify… | Please select all that apply - you can choose more than one answer. | | Reliability *α* = .68  Exploratory Factor analysis found 2 latent factors (the school’s poor implementation capacity and the school health team’s poor functioning)with explained variance 41%. | |
|  | Support needed to improve school mental health promotion | The percentage of schools reported that these supports were needed to improve school mental health. | Would your program benefit from any of the following?   1. Funding 2. Regular teacher training 3. Regular student training 4. Greater school leadership capacity 5. Greater mental health literacy of teachers 6. Community linkages 7. Referral pathways 8. Stronger understanding and support from family/parents 9. Other, please specify… | Please select all that apply (you can choose more than one). | | Reliability *α* = .81  Exploratory Factor analysis found 1 latent factor with explained variance 44.37%. | |

**Supplement B. Descriptive data of the reported implementation success**

Table 1. Reported success of implementation of the five key mental health promotion programs in 161 Junior-high schools in Surabaya (Public, Private, Madrasa)

|  | Total (N=161) | | Public SMP (n=63) | | Private SMP (n=74) | | MTs (n=24) | |
| --- | --- | --- | --- | --- | --- | --- | --- | --- |
| Program | M (SD) | 95% CI | M (SD) | 95% CI | M (SD) | 95% CI | M (SD) | 95% CI |
| UKS ^a,b,d^ | 3.60 (.68) | 3.50- 3.71 | 3.80 (.54) | 3.66-3.93 | 3.56 (.72) | 3.39-3.73 | 3.21 (.72) | 2.90-3.51 |
| SRA | 2.71 (.58) | 2.62-2.80 | 2.81 (53) | 2.67-2.94 | 2.69 (.59) | 2.55-2.82 | 2.54 (.65) | 2.26-2.82 |
| RK | 2.57 (1.26) | 2.37-2.76 | 2.77 (1.28) | 2.45-3.09 | 2.51(1.26) | 2.21-2.80 | 2.21 (1.21) | 1.70-2.72 |
| KS ^a,c,d^ | 3.48 (.83) | 3.34-3.61 | 3.83 (.52) | 3.69-3.96 | 3.36 (.87) | 3.15-3.56 | 2.92 (1.01) | 2.49-3.35 |
| JI | 3.27 (1.10) | 3.10-3.44 | 3.44 (1.03) | 3.18-3.70 | 3.22(1.12) | 2.96-3.48 | 2.96 (1.16) | 2.47-3.45 |

Notes: UKS= Usaha Kesehatan Sekolah (school health unit). SRA= Sekolah Ramah Anak (child-friendly school). RK= Rapor Kesehatanku (extra-curricular mental health module). KS= Konseling Sebaya (peer counsellor). JI= Jirona (mental health and risk behaviour assessment).

Reported perceived success was assessed by the question, “Has the school managed to deliver the program?” (1 = no implementation, 2 = well below standard (<50%), 3 = below standard (50-79%), 4 = standard (80-100%), 5 = exceeding the standard). M = the mean of reported perceived success. SD = Standard deviation.

^a^ = Using ANOVA, a significant mean difference was found among the three school types. ^b^ = Tukey HSD and Bonferroni post-hoc comparisons were also performed and found that public SMPs scored significantly higher than MTs, and ^c^ = Public SMPs scored significantly higher than private SMP and MTs. ^d^ = MTs scored significantly lower than public and private SMPs.

**Supplement C. Cluster analysis on perceived implementation success**

Table 1. The two clusters solution after all perceived implementation success variables were centred

|  | Cluster | |
| --- | --- | --- |
|  | 1 | 2 |
| UKS | 3.18 | 3.85 |
| SRA | 2.35 | 2.93 |
| RK | 1.50 | 3.18 |
| KS | 2.92 | 3.80 |
| JI | 2.30 | 3.84 |

Note: N= 161. UKS= Usaha Kesehatan Sekolah (school health unit). SRA= Sekolah Ramah Anak (child-friendly school). RK= Rapor Kesehatanku (my health report). KS= Konseling Sebaya (peer counsellor). JI= Jirona (mental health and risk behaviour assessment). The scores were the averages of reported perceived success after all variables were centred.


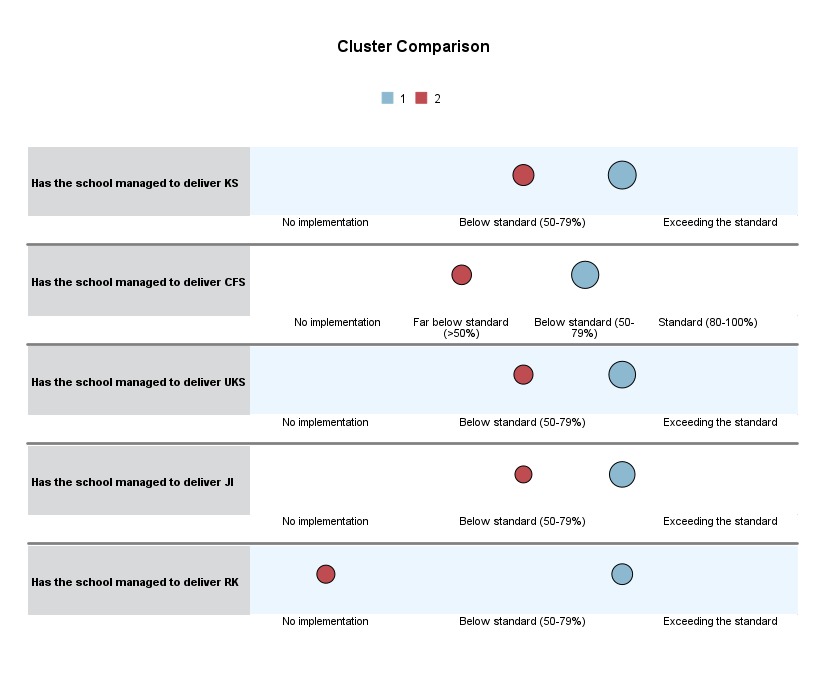


Figure 1. Visual inspection by using 2 cluster comparison on UKS, CFS, RK, KS, JI


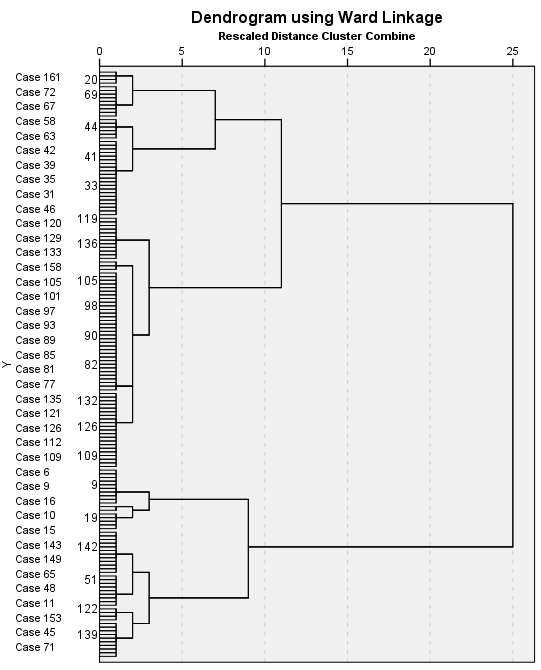


Figure 2. The dendrogram on UKS, CFS, RK, KS, JI

Table 2. Cross-tabulation between perceived implementation success with enablers and barriers to implementation

|  |  | Perceived implementation success | | | |  |  |  |
| --- | --- | --- | --- | --- | --- | --- | --- | --- |
| Factors |  | Poor performing | | Standard performing | | N Total | *Χ*^2^ | Φ |
|  |  | N | % | N | % |  |  |  |
| **Enablers** |  |  |  |  |  |  |  |  |
| Have a school mental health promotion team | Yes | 24 | 40 | 62 | 61.4 | 86 | 6.91* | .20 |
|  | No | 36 | 60 | 39 | 38.6 | 75 |  |  |
|  | Total | 60 | 100 | 101 | 100 | 161 |  |  |
| Policies and supports that are in place | Yes | 23 | 38.3 | 46 | 45.5 | 69 | .80 | .70 |
|  | No | 37 | 61.7 | 55 | 54.5 | 92 |  |  |
|  | Total | 60 | 100 | 101 | 100 | 161 |  |  |
| Partnership with the community health centre | Yes | 47 | 78.3 | 87 | 86.1 | 134 | 1.64 | .10 |
|  | No | 13 | 21.7 | 14 | 13.9 | 27 |  |  |
|  | Total | 60 | 100 | 101 | 100 | 161 |  |  |
| NGOs provide significant support | Yes | 9 | 15 | 20 | 19.8 | 29 | .58 | .06 |
|  | No | 51 | 85 | 81 | 80.2 | 132 |  |  |
|  | Total | 60 | 100 | 101 | 100 | 161 |  |  |
| Support from agencies in the community | Yes | 22 | 36.7 | 48 | 47.5 | 70 | 1.80 | .10 |
|  | No | 38 | 63.3 | 53 | 52.5 | 91 |  |  |
|  | Total | 60 | 100 | 101 | 100 | 161 |  |  |
| Access teacher training in mental health | Yes | 9 | 15 | 40 | 39.6 | 49 | 10.76 | .26 |
|  | No | 51 | 85 | 61 | 60.4 | 112 |  |  |
|  | Total | 60 | 100 | 101 | 100 | 161 |  |  |
| **Barriers** |  |  |  |  |  |  |  |  |
| No school health team | Yes | 15 | 25 | 12 | 11.9 | 27 | 4.64* | -.17 |
|  | No | 45 | 75 | 89 | 88.1 | 134 |  |  |
|  | Total | 60 | 100 | 101 | 100 | 161 |  |  |
| No school mental health team | Yes | 30 | 50 | 31 | 30.7 | 61 | 5.96* | -.19 |
|  | No | 30 | 50 | 70 | 69.3 | 100 |  |  |
|  | Total | 60 | 100 | 101 | 100 | 161 |  |  |
| No relationship with community health centre | Yes | 3 | 5 | 12 | 11.9 | 15 | 2.11 | .11 |
|  | No | 57 | 95 | 89 | 88.1 | 146 |  |  |
|  | Total | 60 | 100 | 101 | 100 | 161 |  |  |
| UKS is not optimally functional | Yes | 28 | 46.7 | 26 | 25.7 | 54 | 7.39** | -.21 |
|  | No | 32 | 53.3 | 75 | 74.3 | 107 |  |  |
|  | Total | 60 | 100 | 101 | 100 | 161 |  |  |
| School lacks mental health literacy | Yes | 22 | 36.7 | 30 | 29.7 | 52 | .83 | -.07 |
|  | No | 38 | 63.3 | 71 | 70.3 | 109 |  |  |
|  | Total | 60 | 100 | 101 | 100 | 161 |  |  |
| Lack of access to teacher training | Yes | 41 | 68.3 | 62 | 61.4 | 103 | .78 | -.07 |
|  | No | 19 | 31.7 | 39 | 38.6 | 58 |  |  |
|  | Total | 60 | 100 | 101 | 100 | 161 |  |  |
| Students are not accessing support services | Yes | 15 | 25 | 17 | 16.6 | 32 | 1.57 | -.10 |
|  | No | 45 | 75 | 84 | 83.4 | 129 |  |  |
|  | Total | 60 | 100 | 101 | 100 | 161 |  |  |
| Parents' disapproval of school mental health | Yes | 0 | 0 | 1 | 1 | 1 | .58 | .06 |
|  | No | 60 | 100 | 100 | 100 | 160 |  |  |
|  | Total | 60 | 100 | 101 | 100 | 161 |  |  |
| Parents do not support referral services | Yes | 8 | 13.6 | 18 | 17.8 | 26 | .50 | .05 |
|  | No | 52 | 86.4 | 83 | 82.2 | 135 |  |  |
|  | Total | 60 | 100 | 101 | 100 | 161 |  |  |
| Teacher’s poor mental health literacy | Yes | 11 | 18.6 | 19 | 18.8 | 30 | .00 | .00 |
|  | No | 49 | 81.4 | 82 | 81.2 | 131 |  |  |
|  | Total | 60 | 100 | 101 | 100 | 161 |  |  |
| The pandemic changed school health program | Yes | 21 | 35 | 30 | 29.7 | 51 | .48 | -.05 |
|  | No | 39 | 65 | 71 | 70.3 | 110 |  |  |
|  | Total | 60 | 100 | 101 | 100 | 161 |  |  |

Note: *Chi-square is significant at *p* < 0.05, ** *p* < 0.01.

**Supplement D.** **Factor analysis on Barriers and enablers**


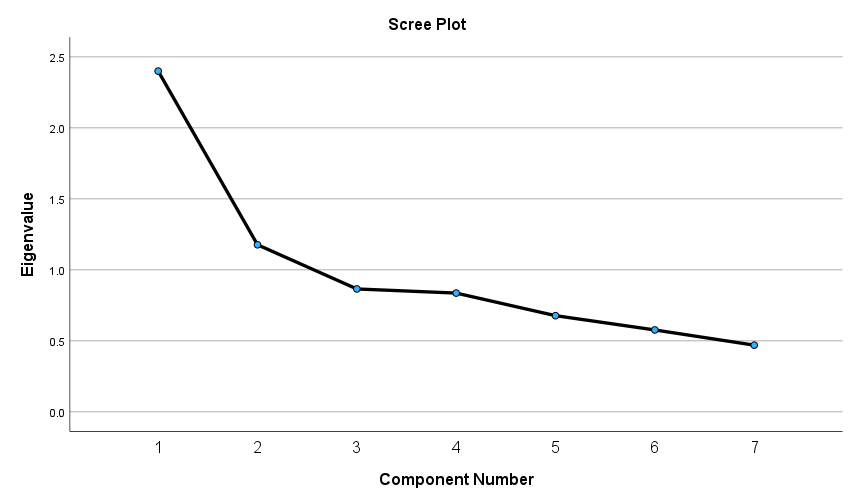


Figure 1. Scree plot for enablers

Table 1. Rotated component matrix for enablers

|  | Component | |
| --- | --- | --- |
|  | 1 | 2 |
| School has a functional school health team | .723 |  |
| Regulations and facilities supported by governments |  | .553 |
| Working partnership with Puskesmas |  | .749 |
| NGO support |  | .487 |
| School can access public services and support from communities |  | .743 |
| ‎Access to mental health training for teachers | .753 |  |
| DoE facilitated training for teachers | .708 |  |
| Extraction Method: Principal Component Analysis.  Rotation Method: Varimax with Kaiser Normalization. | | |


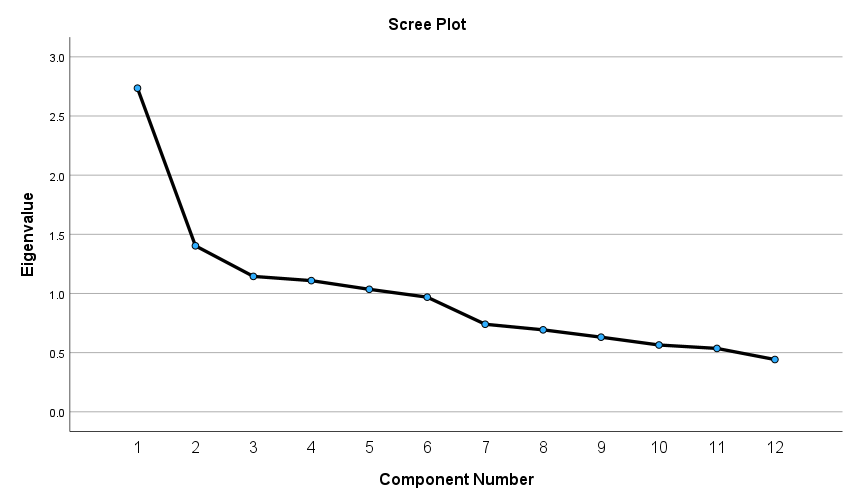


Figure 2. Scree plot for barriers

Table 2. Rotated component matrix for barriers

|  | Component | |
| --- | --- | --- |
|  | 1 | 2 |
| No school health team |  | .747 |
| No school mental health team |  | .380 |
| No functional partnerships with Puskesmas |  | .626 |
| UKS is yet optimally functioning |  | .704 |
| School's poor capacity for dealing with mental health problems | .535 |  |
| Lack of teacher's mental health training | .563 |  |
| Lack of trust from student for accessing services at school | .563 |  |
| Parents don’t follow referral | .690 |  |
| Teachers' poor mental health literacy | .698 |  |
| Pandemic holds back implementation | .538 |  |
| Extraction Method: Principal Component Analysis.  Rotation Method: Varimax with Kaiser Normalization. | | |
